# The 2023’s Growing Evidence Confirming the Relationship between Vitamin D and Autoimmune Diseases

**DOI:** 10.3390/nu15224760

**Published:** 2023-11-13

**Authors:** Maurizio Cutolo, Emanuele Gotelli

**Affiliations:** Laboratory of Experimental Rheumatology and Academic Division of Clinical Rheumatology, Department of Internal Medicine and Specialties, University of Genova, 16132 Genova, Italy

The second Special Issue of Nutrients dedicated to “Vitamin D, Immune Response, and Autoimmune Diseases” will include original data and recent achievements from authors who would like to participate in this research topic.

Vitamin D_3_ (cholecalciferol) is a secosteroid hormone that derives mostly from exposure of the body to the sun and photoconversion of cutaneous 7-dehydrocholesterol, as well as, but to a much lesser extent, from foods rich in Vitamin D [1]. 

Cholecalciferol is usually hydroxylated by hepatic cytochromes (CYP2R1) into calcifediol [25(OH)D_3_], and a second hydroxylation into calcitriol [1,25(OH)_2_D_3_] is catalyzed by CYP27B1. When the latter hydroxylation occurs in the kidney, calcitriol plays a metabolic role by interacting with the vitamin D receptor (VDR) on intestinal and parathyroid cells, regulating serum concentrations of calcium and phosphates.

CYP27B1 is also expressed by innate and adaptive immune cells; in this case, calcitriol acts in a paracrine/autocrine way on VDR expressed by immune cells. The immune effect exerted by calcitriol is the downregulation of nuclear transcription factor NF-kB and, subsequently, of pro-inflammatory cytokines, such as interleukin (IL)-1, IL-6, IL-12, IL-23, and IL-17, and tumor necrosis factor (TNF)-α. Downregulation of these cytokines, therefore, reduces the functional M1 polarization of monocytes/macrophages (secretion of interferon-gamma, IL-1, IL-6, and reactive oxygen species) towards an M2 polarization (release of anti-inflammatory cytokine IL-10) and the polarization of lymphocytes T helper type 1 and 17 (pro-inflammatory) towards a Th2 phenotype (reparative and anti-inflammatory) [1].

Therefore, immune effects of calcitriol in case of deficiency (<30 mg/dL) or adequate (>30 mg/dL) 25(OH)D_3_ serum concentrations have been investigated for more than two decades both in animal and human models of autoimmune inflammatory diseases, with interesting observations noted in 2023 [2].

A comprehensive review has reported that insufficient 25(OH)D_3_ serum concentrations are frequent and associated with unfavorable outcomes in the course of many autoimmune rheumatic musculoskeletal diseases (RMDs), including rheumatoid arthritis (RA), psoriatic arthritis (PsA), systemic lupus erythematosus (SLE), Sjögren’ syndrome (SjS) and others [2]. Also, in pediatric RMDs, an updated review reported significantly lower 25(OH)D_3_ serum concentrations associated with higher pro-inflammatory cytokine concentrations in juvenile idiopathic arthritis, juvenile systemic scleroderma, juvenile SLE, and Behcet’s disease [3].

According to previous studies [1], a cohort of 101 adult patients diagnosed with SLE demonstrated a significant correlation between hypovitaminosis D and high levels of inflammatory activity. This association was evaluated by serum markers (erythrocyte sedimentation rate (ESR), C-reactive protein (CRP), and IL-6). Furthermore, previous studies have found a connection between hypovitaminosis D and the SLE Disease Activity Index and the severity of organ damage estimated by the Damage Index [4].

During SLE, a potential therapeutic role for vitamin D replacement has been postulated in vitro [5]. DNA-containing circulating immune complexes from SLE patients were purified, absorbed, and then consumed by myeloid dendritic cells of healthy subjects, increasing the release of TNF-α and the downregulation of IL-10. On the other hand, co-culture of these myeloid dendritic cells with calcitriol showed opposite effects on TNF-α (downregulation) and IL-10 (upregulation), suggesting a role for calcitriol in mitigating inflammation also in vivo [5].

Hypovitaminosis D has been associated with some features of SjS, such as leukopenia, peripheral neuropathy, and lymphoma [2]. Two genome-wide association studies with the Mendelian randomization approach found opposite results, as higher 25(OH)D_3_ serum concentrations were associated with a reduction of the risk of primary SjS, even if any causal effects of hypovitaminosis D on SjS risk (and vice versa) were identified [6,7].

A systematic review and meta-analysis of 1049 incident RA cases and 15,604 participants did not reveal an association between 25(OH)D_3_ serum concentrations and RA risk [8]. However, in an in vitro wound healing model in RA patients, calcitriol significantly reduced the migration of synovial cells and mesenchymal stromal cells of the joints [9]. Antimigratory and antiproliferative effects of calcitriol were found equal to glucocorticoids tested in the study (dexamethasone, methylprednisolone acetate, and betamethasone) and might confirm the steroidal origin and presence of commonly selected biological actions for both molecules [9].

In a murine model of RA (TNF-transgenic mice), calcitriol downregulated the pro-inflammatory M1 polarization of monocytes/macrophages (evaluated by expression of CD80, IL-6, CXCL10, IFIT1, IFI27, and IF44) in arthritic joints through upregulation of fructose-1,6-biphosphate (FPB1), promoting double-stranded RNA-dependent protein kinase R (PKR) ubiquitination degradation [10].

Moreover, in two different Iranian and Saudi Arabia cohorts (92 and 102 RA patients, respectively), hypovitaminosis D was significantly correlated with RA disease activity scores (DAS), like DAS28-CRP [11] or DAS28-ESR [12], as reported in previous studies [1].

A Mendelian randomization analysis of the population of UK Biobank (332,984 participating, of which 12,774 were affected by autoimmune disorders and 11,164 by autoinflammatory disorders) recently supported a genetic causal link between low 25(OH)D_3_ serum concentrations and the risk of psoriasis (odds ratio 0.91, *p* = 0.005) [13].

A systematic review with a meta-analysis of 25(OH)D_3_ serum concentrations of 1876 psoriatic patients and 7532 controls confirmed that hypovitaminosis D is more common in psoriasis than healthy conditions (paper published in the first edition of this Special Issue) [14].

Similarly, a systematic review with a meta-analysis of 25(OH)D_3_ serum concentrations of 264 patients with psoriatic arthritis (PsA) and 287 healthy controls showed that PsA patients have hypovitaminosis D more frequently than general populations [15].

In a retrospective study involving 233 PsA patients, 25(OH)D_3_ ≤ 20 ng/dL has been associated with a higher discontinuation rate of both conventional disease-modifying antirheumatic drugs (cDMARD (methotrexate), *p* = 0.02) and first biological DMARDs (*p* = 0.02) when compared to 25(OH)D_3_ > 20 ng/dL [16].

When compared to the general population, autoimmune endocrine diseases such as Hashimoto’s thyroiditis (HT) hypovitaminosis D were associated with higher serum concentrations of pro-inflammatory cytokines (IL-1β, IL-6, IL-8, and TNFα) [17,18].

The clinical impact of administering 2800 IU per day of vitamin D3 as an adjunct treatment to the antithyroid drug for approximately 36 months was assessed in a multicenter, double-blinded, randomized placebo-controlled trial in patients with Grave’s disease (autoimmune hyperthyroidism). The trial results did not demonstrate statistically significant clinical benefits [19]. 

In autoimmune type 1 diabetes, inadequate 25(OH)D_3_ serum concentrations have been identified as a major risk factor for the development of the disease, together with specific polymorphisms of the VDR (FokI-FF, Bsml-B, and Apal-A alleles) in a South Indian population [20].

Mendelian randomization studies recently investigated the relationship between vitamin D_3_ and the risk of autoimmune neurological disease. No causal relationship has been identified between hypovitaminosis D and myasthenia gravis (MG) [21]; a systematic review with a meta-analysis including 219 MG patients and 231 healthy controls showed significantly lower 25(OH)D_3_ serum concentrations in MG patients than the general population [22].

On the contrary, Mendelian randomization analysis found hypovitaminosis D as a clear risk factor for developing multiple sclerosis (MS) [23].

A recent review resumed immunomodulating effects of vitamin D_3_ in the course of MS: promotion of oligodendrocyte proliferation and differentiation, enhancement of neurotrophins expression (brain-derived neurotrophic factor, ciliary neurotrophic factor, glial cell line-derived neurotrophic factor, and nerve growth factor), reduction of pro-inflammatory activation of microglia, reactive astrogliosis, and oxidative stress and stabilization of the blood–brain barrier (paper published in the first edition of this Special Issue) [24].

In relapsing–remitting MS, a systematic review with a meta-analysis of nineteen clinical studies found weak pieces of evidence that vitamin D supplementation added to standard therapy could participate in the prevention of the clinical relapse of the disease, but of great value, calcitriol significantly reduced the developed of magnetic resonance imaging lesions in the central nervous system of MS patients (paper published in the first edition of this Special Issue) [25].

Moreover, in a murine model of progressive MS, vitamin D supplementation positively affected cortical pathology and neuroaxonal damage by reducing oxidative stress (an immunohistochemistry assessment was conducted in the paper published in the first edition of this Special Issue) [26].

Lastly, vitamin D supplementation has been proposed as an ancillary treatment for COVID-19 (paper published in the first edition of this Special Issue) and long-term neurological symptoms of long-COVID (fatigue, brain fog, anxiety, depression, and sleep disorders). However, there is no conclusive evidence [27,28,29].

In conclusion, the latest evidence on Vitamin D and autoimmune diseases (rheumatological, endocrinological, neurological, and many others) constantly stimulate new research to clarify the pathophysiology of this link. Multicenter, double-blinded, randomized, placebo-controlled trials are still lacking and desirable to determine the best use of vitamin D supplements (dose, duration, intake) as ancillary therapy to improve at least the quality of life of autoimmune patients [30].

As Editors of the second Special Issue on “Vitamin D, Immune Response, and Autoimmune Diseases,” we are extremely excited to receive a contribution that will surely enter a fast and successful development of the recent literature on this crucial topic.

## References

[1] Cutolo M., Smith V., Paolino S., Gotelli E. (2023). Involvement of the secosteroid vitamin D in autoimmune rheumatic diseases and COVID-19. Nat. Rev. Rheumatol..

[2] Athanassiou L., Kostoglou-Athanassiou I., Koutsilieris M., Shoenfeld Y. (2023). Vitamin D and Autoimmune Rheumatic Diseases. Biomolecules.

[3] Stawicki M.K., Abramowicz P., Sokolowska G., Wołejszo S., Grant W.B., Konstantynowicz J. (2023). Can vitamin D be an adjuvant therapy for juvenile rheumatic diseases?. Rheumatol. Int..

[4] Shevchuk S., Marynych L., Malovana T., Denyshchych L. (2023). Vitamin D level in patients with systemic lupus erythematosus: Its relationship to disease course and bone mineral density. Lupus Sci. Med..

[5] Li M., Luo L., Lin C., Ni B., Zou L., Song Z., Hao F., Wu Y., Luo N. (2023). Vitamin D3 mitigates autoimmune inflammation caused by activation of myeloid dendritic cells in SLE. Exp. Dermatol..

[6] Zhao S.S., Burgess S. (2023). Vitamin D is associated with reduced risk of Sjögren’s syndrome: A Mendelian randomization study. Rheumatology.

[7] Zhao M., Wei F., Li H., Wang Z., Wang S., Liu Y., Fei G., Ge Y., Wei P. (2023). Serum vitamin D levels and Sjogren’s syndrome: Bi-directional Mendelian randomization analysis. Arthritis Res. Ther..

[8] Clasen J.L., Cole R., Aune D., Sellon E., Heath A.K. (2023). Vitamin D status and risk of rheumatoid arthritis: Systematic review and meta-analysis. BMC Rheumatol..

[9] Huovinen J., Palosaari S., Pesonen P., Huhtakangas J.A., Lehenkari P. (2023). 1,25(OH)_2_D_3_ and its analogue calcipotriol inhibit the migration of human synovial and mesenchymal stromal cells in a wound healing model—A comparison with glucocorticoids. J. Steroid Biochem. Mol. Biol..

[10] Zhu W., Zhu Y., Zhang S., Zhang W., Si Z., Bai Y., Wu Y., Fu Y., Zhang Y., Zhang L. (2023). 1,25-Dihydroxyvitamin D regulates macrophage activation through FBP1/PKR and ameliorates arthritis in TNF-transgenic mice. J. Steroid Biochem. Mol. Biol..

[11] Bajestani F.S., Khajavian N., Salarbashi D., Kafili M., Ashori F., Hajavi J. (2023). Relationship Between Serum Vitamin D Level and Disease Severity in Rheumatoid Arthritis. Clin. Med. Insights: Arthritis Musculoskelet. Disord..

[12] Alharbi S., Alhabib E., Ghunaim R., Alreefi M.M., Alharbi R.A. (2023). Vitamin D Deficiency in Saudi Patients with Rheumatoid Arthritis. Cureus.

[13] Zhao S.S., Mason A., Gjekmarkaj E., Yanaoka H., Burgess S. (2023). Associations between vitamin D and autoimmune diseases: Mendelian randomization analysis. Semin. Arthritis Rheum..

[14] Formisano E., Proietti E., Borgarelli C., Pisciotta L. (2023). Psoriasis and Vitamin D: A Systematic Review and Meta-Analysis. Nutrients.

[15] Radić M., Đogaš H., Kolak E., Gelemanović A., Nenadić D.B., Vučković M., Radić J. (2023). Vitamin D in psoriatic arthritis—A systematic review and meta-analysis. Semin. Arthritis Rheum..

[16] Rotondo C., Cantatore F.P., Cici D., Erroi F., Sciacca S., Rella V., Corrado A. (2023). Vitamin D Status and Psoriatic Arthritis: Association with the Risk for Sacroiliitis and Influence on the Retention Rate of Methotrexate Monotherapy and First Biological Drug Survival—A Retrospective Study. Int. J. Mol. Sci..

[17] Siddiq A., Naveed A.K., Ghaffar N., Aamir M., Ahmed N. (2023). Association of Pro-Inflammatory Cytokines with Vitamin D in Hashimoto’s Thyroid Autoimmune Disease. Medicina.

[18] Czarnywojtek A., Florek E., Pietrończyk K., Sawicka-Gutaj N., Ruchała M., Ronen O., Nixon I.J., Shaha A.R., Rodrigo J.P., Tufano R.P. (2023). The Role of Vitamin D in Autoimmune Thyroid Diseases: A Narrative Review. J. Clin. Med..

[19] Grove-Laugesen D., Ebbehoj E., Watt T., Riis A.L., Østergård T., Bruun B.J., Christiansen J.J., Hansen K.W., Rejnmark L. (2023). Effect of Vitamin D Supplementation on Graves’ Disease: The DAGMAR Trial. Thyroid®.

[20] Thirunavukkarasu R., Chitra A., Asirvatham A., Jayalakshmi M. (2023). Association of Vitamin D Deficiency and Vitamin D Receptor Gene Polymorphisms with Type 1 Diabetes Risk: A South Indian Familial Study. J. Clin. Res. Pediatr. Endocrinol..

[21] Fan Y., Huang H., Chen X., Chen Y., Zeng X., Lin F., Chen X. (2023). Causal effect of vitamin D on myasthenia gravis: A two-sample Mendelian randomization study. Front. Nutr..

[22] Bonaccorso G. (2023). Myasthenia Gravis and Vitamin D Serum Levels: A Systematic Review and Meta-analysis. CNS Neurol. Disord. Drug Targets.

[23] Fazia T., Baldrighi G.N., Nova A., Bernardinelli L. (2023). A systematic review of Mendelian randomization studies on multiple sclerosis. Eur. J. Neurosci..

[24] Sangha A., Quon M., Pfeffer G., Orton S.-M. (2023). The Role of Vitamin D in Neuroprotection in Multiple Sclerosis: An Update. Nutrients.

[25] Langlois J., Denimal D. (2023). Clinical and Imaging Outcomes after Vitamin D Supplementation in Patients with Multiple Sclerosis: A Systematic Review. Nutrients.

[26] Haindl M.T., Üçal M., Wonisch W., Lang M., Nowakowska M., Adzemovic M.Z., Khalil M., Enzinger C., Hochmeister S. (2023). Vitamin D—An Effective Antioxidant in an Animal Model of Progressive Multiple Sclerosis. Nutrients.

[27] Gotelli E., Soldano S., Hysa E., Paolino S., Campitiello R., Pizzorni C., Sulli A., Smith V., Cutolo M. (2022). Vitamin D and COVID-19: Narrative Review after 3 Years of Pandemic. Nutrients.

[28] Gotelli E., Soldano S., Hysa E., Casabella A., Cere A., Pizzorni C., Paolino S., Sulli A., Smith V., Cutolo M. (2023). Understanding the Immune-Endocrine Effects of Vitamin D in SARS-CoV-2 Infection: A Role in Protecting against Neurodamage. Neuroimmunomodulation.

[29] Cutolo M., Sulli A., Smith V., Gotelli E. (2023). Emerging nailfold capillaroscopic patterns in COVID-19: From acute patients to survivors. Reumatismo.

[30] Talarico R., Askanase A.D., Cutolo M. (2023). Editorial: Psychological impact and quality of life in rheumatic and musculoskeletal diseases. Front. Med..

